# Serotonin circuits and anxiety: what can invertebrates teach us?

**DOI:** 10.1007/s10158-012-0140-y

**Published:** 2012-08-24

**Authors:** Kevin P. Curran, Sreekanth H. Chalasani

**Affiliations:** Molecular Neurobiology Lab, The Salk Institute for Biological Studies, 10010 N. Torrey Pines Road, La Jolla, CA 92037 USA

**Keywords:** Serotonin, Fear, Anxiety disorder, *D. melanogaster*, *C. elegans*

## Abstract

Fear, a reaction to a threatening situation, is a broadly adaptive feature crucial to the survival and reproductive fitness of individual organisms. By contrast, anxiety is an inappropriate behavioral response often to a perceived, not real, threat. Functional imaging, biochemical analysis, and lesion studies with humans have identified the HPA axis and the amygdala as key neuroanatomical regions driving both fear and anxiety. Abnormalities in these biological systems lead to misregulated fear and anxiety behaviors such as panic attacks and post-traumatic stress disorders. These behaviors are often treated by increasing serotonin levels at synapses, suggesting a role for serotonin signaling in ameliorating both fear and anxiety. Interestingly, serotonin signaling is highly conserved between mammals and invertebrates. We propose that genetically tractable invertebrate models organisms, such as *Drosophila melanogaster* and *Caenorhabditis elegans,* are ideally suited to unravel the complexity of the serotonin signaling pathways. These model systems possess well-defined neuroanatomies and robust serotonin-mediated behavior and should reveal insights into how serotonin can modulate human cognitive functions.

## Introduction

Organisms develop mechanisms to adapt to their changing environment (Tinbergen 1963; Seligman 1970; Mayr 1974). They use exquisitely designed sensory systems to receive environmental cues and respond appropriately, favoring cues that aid survival and reproduction, while avoiding those that indicate danger (Hollis 1982; Domjan 2005). These complex and often energetically costly sensory systems (e.g., olfactory, gustatory, visual) are typically coupled with downstream machinery (e.g., cognitive, hormonal, motor output) in order to take full advantage of environmental changes (Ames and Li et al. 1992; Attwell and Laughlin 2001; Lennie 2003; Niven et al. 2003; Nawroth et al. 2007; Niven et al. 2007). Perhaps the most crucial of these functions is the ability to recognize a threat: an increase in temperature, a poisonous fruit, or the scent of a predator. Individuals that fail to detect and avoid threats are likely to experience injury or death; thus, the machinery governing threat response must be precise (Plutchik 1980).

Fear and anxiety are complex behaviors that represent responses to environmental threats. These two behaviors differ in that fear is a response to a real or clearly identifiable threat and functions to remove the individual from a harmful situation (Belzung and Philippot 2007). In contrast, anxiety is a contrived or exaggerated fear response (Chaffey et al. 2002) and often proceeds in the absence of a truly threatening stimulus. This important distinction has led most psychologists and evolutionary biologists to regard fear as an appropriate and adaptive response (Barlow and Durand 2011), and anxiety as psychologically unhealthy and often associated with unwarranted physiological stress across multiple organ systems (Kessler et al. 2005).

In this review, we first briefly summarize the history and biology of anxiety-related disorders; we then describe how invertebrates, with their powerful genetic tools and relatively simple neurocircuitry, can contribute to our understanding of anxiety-related ailments. Of course, an inherent challenge with the use of an invertebrate model system to analyze a human emotional behavior is that organisms such as fruit flies and nematodes do not possess the full suite of characters that comprise human emotion (Tulving 2001). Most notably, invertebrates are missing a sophisticated sense of self-awareness, termed “autonoetic consciousness”. This heightened sense of self facilitates the full experience of human behavior by mentally placing an individual in the past, the future and in possible alternative situations as the threatening stimulus is processed (Tulving 1987; Gardiner 2001; Lou et al. 2004). There is currently no compelling evidence that any non-hominids are in possession of this cognitive ability (Tulving 2001; Cloninger 2009). However, if an emotional behavior is reduced down to its individual components, one finds that certain components of the behavior are accessible in simple model systems. For example, psychologists largely agree that anxiety is composed of three steps: (1) perception and appraisal of a threatening stimulus, (2) cognitive and physiological processing of the stimulus, and (3) specific behavioral output (including the fight or flight response) (Arnold 1960; Scherer 1984; Lowe and Ziemke 2011). In the final section of this review, we describe recent work in the nematode, *Caenorhabditis elegans*, which lends to a circuit level understanding of how a threatening stimulus is sensed, and studies in the fruit fly, *Drosophila melanogaster*, which describes how a behavioral response to a threat (fighting) is generated.

## Anxiety: a historical perspective

The Greeks may have been the first civilization to formally recognize anxiety-related disorders as a medical condition (Gabriel 1987; Hunt 1988). In 332 BC, Aristotle surmised that “vapors” emanating from the heart and brain could induce a “hysteric” or especially nervous condition (Stone 1997). In fact, the word “panic” is derived from the Greek god, Pan, who elicits irrational fear when aroused from his sleep (Stone 1997). The earliest recorded attempts to ameliorate anxiety symptoms used sedatives and electrical stimulation. In the first century AD, patients suffering from anxiety were sedated using opium (Shorter 1997). A hundred years later, Claudius Galen, a prominent Roman physician/philosopher (131–201 AD), applied shocks from electric eels to remedy anxiety-related ailments with modest success (Stillings 1975).

Medical advances during the nineteenth century allowed anatomists to correlate specific lesions in brain-damaged patients to a cognitive or emotional deficiency, a process known as functional localization (Kandel et al. 2000). In 1861, Pierre Paul Broca used this technique to correctly link the capacity to articulate speech with a confined portion of the cortex, the inferior frontal gyrus, now termed “Broca’s area” (Broca 1861). In 1937, James Papez, a Cornell University anatomist, proposed an influential theory of how emotional behaviors are generated and, most importantly, identified a neural pathway driving these behaviors, the Papez circuit (Papez 1995). Papez suggested that emotions are invoked via neural pathways beginning with sensory information and integrated hypothalamal and cortical stimulation (LeDoux 1996). Subsequent neuroanatomical findings have modified this circuit; however, the broader concept of integrated neural pathways underpinning emotional behavior remains intact (Bear 2006).

## HPA axis, amygdala, and serotonin: key players driving mammalian fear and anxiety behaviors

**Fig. 1:**
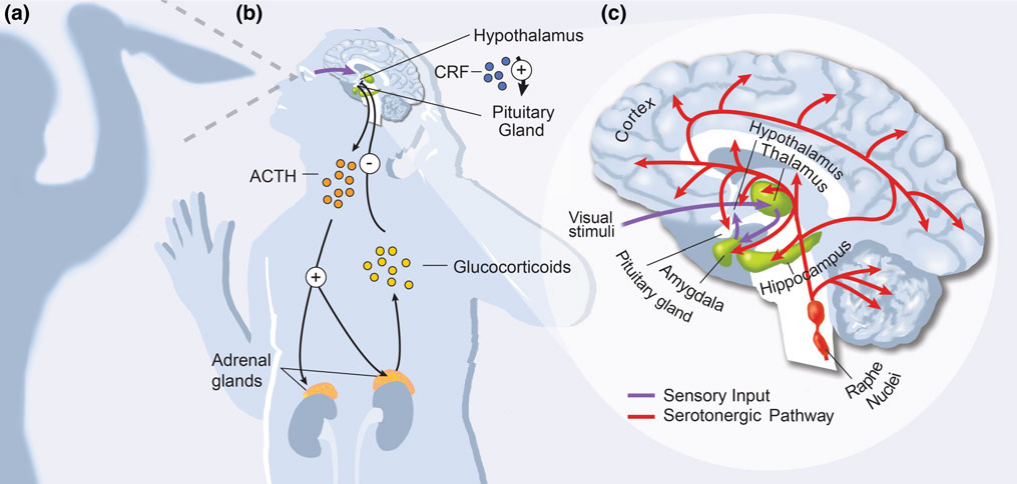
Neurobiology of mammalian fear/anxiety response. **a** Threatening stimulus approaches the subject. A *fearful* reaction occurs in response to a definite threat: a real attacker wielding a knife. An *anxious* response occurs in response to an imprecise or unknown threat: a dark shadow reminiscent of an attacker (Kaplan et al. 1998; Barlow 2002). **b** Physiological processing of threatening stimulus. Distressing visual information enters the retina, is processed by visual circuitry, and activates the hypothalamus (Shi and Davis 2001; Davis 2006). Once engaged, the hypothalamus initiates the HPA axis by releasing CRF (corticotrophin-releasing factor). In response, the pituitary releases ACTH (adrenocorticotrophin-releasing hormone) into the bloodstream, which prompts the adrenal gland to release glucocorticoids (ex. cortisol). The glucocorticoids bind receptors on the hypothalamus and pituitary, preventing further release of CRF and ACTH (Mathew et al. 2008). **c** Enlarged view of neurological processing of threatening stimulus. *Amygdala processing* (*purple arrows*): Visual data captured by the retina is first processed by the thalamus, which in turn innervates the amygdala (*green* regions). The amygdala integrates memory information from the hippocampus and context and autonoetic consciousness from the cortex (LeDoux 2000; Zald 2003; Phelps and LeDoux 2005) (e.g., orbitofrontal cortex, anterior cingulate cortex). Additionally, the amygdala reciprocally innervates various brain regions including locus coeruleus, BNST (bed nucleus of the stria terminalis), anterior insula, and hypothalamus (Paulus and Stein 2006; Mathew et al. 2008) (regions not shown). *Serotonergic circuit* (*red arrows*): Serotonin is released from pre-synaptic neurons within the dorsal and median raphe nuclei on the midline of the brainstem. Serotonergic neurons innervate regions of the cerebellum, thalamus, hippocampus, hypothalamus, basal ganglia, frontal cortex, and amygdala (Kandel et al. 2000)

Our current understanding of the neurobiology of fear and anxiety centers largely on three interrelated systems: the amygdala, the HPA (hypothalamic–pituitary–adrenal gland) axis, and neuromodulators (e.g., serotonin) (Fig. 1). Each of these systems will be briefly discussed; however, this review will largely focus on the role of serotonin in modulating these behaviors.

Information encoding a threatening stimulus enters an individual’s brain, undergoes context-dependent processing through a sensory neural circuit, and is eventually relayed to the hypothalamus (Shi and Davis 2001; Davis 2006). Once activated, the hypothalamus engages the HPA axis: a hormonal feedback system between two brain regions (hypothalamus and pituitary) and the adrenal gland (a small organ on the top of the kidneys) (Fig. 1b). The HPA axis regulates the bodily reaction to stress and constitutes an important facet of fearful or anxious behavior (Jacobson 2005). The neurochemical component of fearful/anxious HPA signaling begins when stressful stimuli prompt the hypothalamus to release the neuropeptide CRF (corticotrophin-releasing factor). CRF release triggers the proximally located pituitary to release ACTH (adrenocorticotrophin-releasing hormone) into the bloodstream (Jacobson 2005). The adrenal cortex detects the increase of ACTH in the bloodstream and, in turn, triggers the adrenal gland to release glucocorticoids, including cortisol. A negative feedback loop is completed when glucocorticoids bind the glucocorticoid receptors on the hypothalamus and pituitary, thereby suppressing further release of CRF and ACTH (Mathew et al. 2008). Abnormalities in these neurochemical HPA interactions are implicated in multiple stress-related disorders, including anxiety (Laue et al. 1991; Licinio et al. 1996; De Kloet et al. 1998; Pascual 2003).

The amygdala, an almond shaped cluster of nuclei within the medial temporal lobes of complex vertebrate brains, is also critical for generating emotional fear and anxiety behaviors (Kandel et al. 2000; Phelps and LeDoux 2005). The amygdala receives inputs from the sensory systems (e.g., auditory, visual) via the thalamus and integrates these stimuli with cortical processing (context, memory, and conscious self-regulation**)** before generating efferent signals that contribute to the threat response (Fig. 1c) (Gray 1999; Phelps and LeDoux 2005; Shin et al. 2006; Hariri and Whalen 2011; Johansen et al. 2011). Correlative experiments linking functional imaging in the brain with fear conditioning emphasize the key role played by the amygdala in fear and anxiety behaviors. Fear conditioning, or Pavlovian fear conditioning, is a form of learning in which a neutral stimulus (conditioned stimulus) elicits fearful or anxious behavior after association with an aversive event (unconditioned stimulus) (Pavlov 1927). LeDoux and others surmise these two stimuli (conditioned and unconditioned) are integrated within the amygdala circuitry via synaptic plasticity and the Hebbian processes (Johansen et al. 2011).

**Fig. 2:**
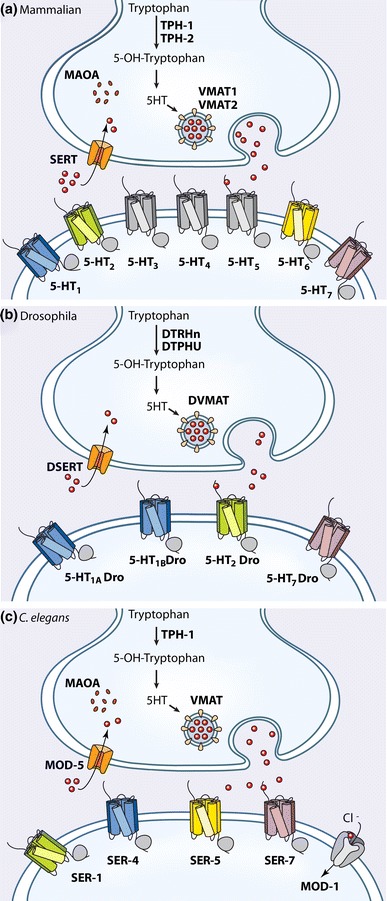
Conservation of serotonin pathway machinery. **a** Mammalian serotonin pathway. *TPH* Tryptophan hydroxylase synthesizes Serotonin (5-HT) from tryptophan. There are 2 mammalian TPH isoforms: TPH-1 and TPH-2 (Walther al. 2003). The *VMAT* vesicular monoamine transporter pumps 5-HT from the cytoplasm into either small synaptic vesicles or dense core vesicles (Liu and Edwards 1997). There are 2 mammalian VMAT proteins: VMAT1, found in neuroendocrine cells, and VMAT2, expressed in all CNS serotonergic neurons (Erickson et al. 1992; Weihe et al. 1994). Post-synaptic neurons express 7 classes of serotonin receptors. 5-HT_1_, 5-HT_2_, 5-HT_4_, 5-HT_5_, 5-HT_6_, 5-HT_7_ are *GPCR G*-protein-couple receptors (Hartig 1997). 5-HT_3_ is a ligand-gated sodium channel with no orthologue in the invertebrates (Hanna et al. 2000). *SERT* Serotonin transporter protein removes 5-HT from the synaptic cleft (Chang et al. 1996). 5-HT is degraded by *MAO* monoamine oxidase, which catalyzes oxidative deamination of 5-HT. There are 2 forms: MAO A and MAO B (Bach et al. 1988). **b** *D. melanogaster* serotonin pathway. Serotonin (5-HT) is synthesized by 2 tryptophan hydroxylase homologues: DTRHn (hydroxylates tyryptophan) and DTPHu (hydroxylates both tryptophan and phenylalanine) (Neckameyer and White 1992; Neckameyer et al. 2007). 5-HT is packaged into vesicles with DVMAT, homologous to mammalian VMAT1 and VMAT2 (Greer et al. 2005). 4 classes of serotonin receptors have been identified: 5-HT_7_Dro is functionally similar to 5HT_7_ (Becnel et al. 2011). 5-HT_1A_Dro and 5-HT_1B_Dro are most functionally similar to 5HT_1_ (Gerhardt et al. 1996). 5-HT_2_Dro is related in structure and pharmacology to 5-HT_2_ (Colas et al. 1995). 5-HT is removed from the synaptic cleft by DSERT, homologous to mammalian SERT (Demchyshyn et al. 1994). **c** *C. elegans* serotonin pathway. Serotonin (5-HT) is synthesized by TPH-1 (Sze et al. 2000). 5-HT is packaged with VMAT, homologous to mammalian VMAT1 and VMAT2 (Duerr et al. 1999). Five serotonin receptors have been identified in *C. elegans*. Four are G-protein coupled receptors (GPCR): SER-4 resembles 5HT_1_ (Olde and McCombie 1997), SER-5 has only sequence similarity to 5-HT_6_ (Hapiak et al. 2009), SER-1, a 5HT_2_-like receptor (Hamdan et al. 1999) and SER-7, homologous to 5-HT_7_ mammalian receptor (Hobson et al. 2003). MOD-1 is a 5-HT gated chloride channel not found in mammals (Ranganathan et al. 2000). 5-HT is removed from the synaptic cleft by MOD-5, a serotonin reuptake transporter (Cooper et al. 1996). MOD-5 is homologous to mammalian SERT (Ranganathan et al. 2001). Once removed from the cleft, 5HT is degraded by an enzyme homologous to monoamine oxidase (Weyler 1992)

To date, pharmaceutical attempts to treat anxiety disorders by modulating neurotransmitter activity have had mixed success. In 1904, Bayer introduced “Veronal”, a barbiturate that soon became the drug of choice in private nervous clinics (Shorter 1997). Barbiturates act mostly by enhancing GABA signaling by binding GABA receptors on target neurons (Nemeroff 2003). Despite its addictive side effect, Veronal’s initial success led to the development of chemically similar anxiolytics (anxiety-reducing compounds). Throughout the past century, chemists have produced various classes of anxiolytics (e.g., beta-blockers, tricyclic anti-depressants, benzodiazepines) in an attempt to improve efficacy while minimizing detrimental side effects (Barlow and Durand 2011). A recent meta-analysis of multiple classes of drug treatments for general anxiety disorder determined that fluoxetine (Prozac), a selective serotonin reuptake inhibitor (SSRI), elicits the highest patient response to treatment (Baldwin et al. 2011). SSRIs block the reuptake of serotonin into the presynaptic nerve terminals, thereby increasing the synaptic concentration of serotonin (Fig. 2a) (Kandel et al. 2000). Of patients suffering from anxiety, 63 % report amelioration of symptoms when treated with Prozac (Baldwin et al. 2011). This high efficacy has made Prozac the primary, first-line treatment for anxiety disorders (Koen and Stein 2011).

## The serotonin pathway

The high success rate of SSRIs strongly suggests a biological link between anxiety and the serotonin pathway; however, the details of this link remain unclear. Serotonin (5HT), a monoamine neurotransmitter, is utilized by most of the animal kingdom as a neural circuit modulator (Hen 1993). In mammals, serotonin modulates a wide range of behaviors, including pain perception, sleep, aggression, feeding, and mood (Hen 1993; Weiger 1997; Carre-Pierrat et al. 2006). Serotonergic neurons originate from the dorsal and median raphe nuclei in the brain stem and project into multiple forebrain and limbic structures (e.g., amygdala, thalamus, hypothalamus, hippocampus, and frontal cortex), thereby constituting a serotonin circuit (Fig. 1c) (Kandel et al. 2000). Fear and anxiety stimuli selectively activate serotonergic neurons in the dorsal raphe nucleus, which then project into the amygdala and the hypothalamic portion of the HPA axis (Goddard and Charney 1997; Lowry 2002; Bauman and Amaral 2005; Spannuth et al. 2011). One model to explain SSRI efficacy reasons that serotonin can suppress the hyperactivation of the amygdala (Harmer et al. 2006; Furmark 2009). While SSRIs might have multiple neural circuit targets, their treatment successes emphasize a crucial role for serotonin in modulating human anxiety-related behavior.

The molecular machinery governing serotonin signaling across neurons is well characterized and also well conserved among vertebrates and invertebrates (Fig. 2). Serotonin is synthesized from tryptophan by tryptophan hydroxylase in the cytoplasm of the presynaptic serotonergic neuron. Vesicle monoamine transporters then package serotonin into vesicles. These vesicles fuse with the cell membrane and the stored neurotransmitter is released into the synaptic cleft and bound by serotonin receptors on the surface of postsynaptic cells. The signal is terminated when unbound serotonin is reabsorbed back into the presynaptic cell by reuptake transporters, thereby limiting the spread of 5HT concentration. Finally, a catabolic enzyme, monoamine oxidase, metabolizes 5HT to non-active aldehyde derivatives (Horvitz et al. 1982; Kandel et al. 2000; Chase and Koelle 2007). Discrepancies in the activity of the serotonin pathway correlate with anxious behaviors. Combining functional imaging with genomic analysis, researchers have associated a short allele of the promoter (5-HTTLPR) for the human serotonin transporter gene (SLC6A4) with anxiety-related personality traits, for example, fear condition-ability (Hariri et al. 2002). Individuals carrying one or two copies of the truncated version of SLC6A4 exhibit reduced serotonin signaling and, interestingly, greater amygdala activity. Consistent with these findings, high-anxiety subjects carrying the short allele of the serotonin transporter promoter, 5-HTTLPR, exhibit elevated amygdala activity during an anxiety-inducing public speaking task compared to carriers of the long allele (Furmark et al. 2004). These studies suggest 5HT can suppress the activity in the amygdala that is relevant to anxiety.

## Serotonin complexity hinders precise understanding

While these studies implicate a strong link between serotonergic circuits and fear and anxiety behavior, less is known about *how* serotonin modulates neural circuits to affect these behaviors (Baldwin et al. 2011; Koen and Stein 2011). Treatments that deplete serotonin levels have produced both anxiety-reducing and anxiety-inducing behavior (Handley 1995). This paradox is further illustrated by the fact that long-term exposure to SSRIs reduces anxiety, while acute exposure (first 2 weeks of treatment) is often accompanied with anxiety-inducing behavior in both humans and animal models (Gordon and Hen 2004; Grillon et al. 2007). Furthermore, it should be noted that the significance of SSRI efficacy, in regard to its antidepressant benefits, is currently being debated. Irving Kirsch and colleagues performed a meta-analysis of published and unpublished data (obtained from the FDA via the Freedom of Information Act) analyzing 35 placebo-controlled trials of four popular SSRI drugs (Fluoxetine, Venlafaxine, Nefazodone, Paroxetine). The study concluded that, compared with placebo, these new-generation SSRI antidepressants do not produce clinically significant improvements in patients with mild or moderate depression, but do show significant effects only in severely depressed patients (Kirsch et al. 2008; Horder et al. 2011). It remains unclear what biological variation generates this multitude of behavioral responses to pharmacological intervention. Taken together, these SSRI-related incongruities emphasize our incomplete understanding of the complexities of serotonin signaling as it pertains to cognitive behaviors.

While functional imaging and genetic analysis of humans have provided a neuroanatomical map of serotonin-modulated behaviors, attempts to elucidate a circuit level understanding of anxious behavior have only been marginally successful due to the cellular complexity of the mammalian brain. This is understandable; the human brain consists of approximately 100 billion interconnected neurons, which are largely inaccessible for analysis (Kandel et al. 2000). Fortunately, the serotonin machinery is largely conserved throughout the animal kingdom (Fig. 2), facilitating the study of this intricate pathway in biological systems that are more tractable than the mammalian brain. Ideally, such a model should be capable of executing sophisticated behaviors that are modulated by serotonin signaling. Small invertebrate model systems such as *D. melanogaster* and *C. elegans* are exceptionally suited to this task (Kaletta and Hengartner 2006; Iliadi 2009; Pungaliya et al. 2009; Dimitriadi and Hart 2010; Kullyev et al. 2010).

## Serotonin signaling in *C. elegans* aversive behavior

The nematode, *C. elegans*, with just 302 neurons connected by identified chemical and electrical synapses (White et al. 1986) is ideally suited to unravel conserved serotonin signaling pathways driving whole animal behavior. In the worm, serotonin is made from 6 neuronal types (HSN, NSM, VC4, VC5, ADF, and CP1-6) by a tryptophan hydroxylase (TPH-1) with strong homology to mammalian TPH-1 (Sze and Victor et al. 2000). Similar to mammalian serotonergic neurons (Fig. 2), a *C. elegans* VMAT packages serotonin into vesicles (Duerr et al. 1999). Also, *C. elegans* neurons express at least five different serotonin receptors: SER-4 (5-HT_1_-like), SER-1 (5-HT_2_-like), SER-5 (5-HT_6_-like), SER-7 (5-HT_7_-like), and MOD-1 (a 5-HT gated Cl- channel not found in mammals) (Olde and McCombie 1997; Hamdan et al. 1999; Ranganathan et al. 2000; Hobson et al. 2003; Hapiak et al. 2009). Moreover, MOD-5, the worm homologue of mammalian SERT, acts to remove serotonin from synapses (Ranganathan et al. 2001). Interestingly, fluoxetine, a commonly used SSRI that blocks mammalian SERT, can also block MOD-5 (*C. elegans* SERT homologue), among other targets in the worm (Kullyev et al. 2010).

In *C. elegans*, serotonin signaling plays a significant role in mediating avoidance to the repellent odorant, octanol (Segalat et al. 1995; Troemel et al. 1997; Sawin et al. 2000). As previously mentioned, the perception and appraisal of a stressful or repellent stimulus is the first component of anxious or fearful behavior in humans. Before an organism can determine an appropriate behavioral output, the threatening stimulus must be accurately processed via the sensory system. As described below, elegant studies in *C. elegans*, which seek to unravel the mechanisms of avoidance behavior, provide insights into serotonin-mediated sensory activity. The studies reveal specific interactions of different serotonin receptors acting in concert with peptidergic and additional monoaminergic pathways (Chao et al. 2004; Harris et al. 2009, 2010; Mills et al. 2012).

The Komunieki laboratory has utilized sensory-mediated avoidance behavior, driven by a well-defined neuronal circuit, to dissect the pathways governing serotonin-mediated stimulus appraisal (Chalfie et al. 1985; Chao et al. 2004; Hilliard et al. 2004). The group has identified cell-specific roles for serotonin receptors facilitating avoidance to 30 % octanol, a *C. elegans* repellent. To administer the repellent, a hair is taped to a toothpick, dipped in 30 % octanol, and placed in front of a worm exhibiting forward sinusoidal locomotion (Harris et al. 2009). Using mutant analysis and cell-specific RNAi knockdown, Harris and colleagues determined that MOD-1, the serotonin gated Cl- channel, is necessary in the AIB interneuron to properly receive sensory input and that SER-1, the G-protein-coupled serotonin receptor, is required in the RIA/ring motor neuron to properly innervate head muscle. Upon determining the repellent was sensed by the ASH amphid sensory neuron but not finding a known serotonin receptor in ASH, the group performed an RNAi knockdown of previously uncharacterized *C. elegans* biogenic amine receptors and identified SER-5 to be essential for the serotonergic modulation of aversive responses to 30 % octanol (Harris et al. 2009). SER-5 is a novel serotonin receptor that, based on sequence similarity and conserved functional motifs, is most closely related to mammalian 5-HT6 receptors. Together, these findings identify multiple points within a neurological circuit from which serotonin can exert control on the response to an aversive agent.

Additional reports from this group have described how peptide signaling and octopamine signaling integrate with this serotonin circuit to provide a dynamic system for modulating sensory-mediated locomotion (Harris et al. 2010). Harris and colleagues found octopamine signaling inhibits the serotonin sensitized ASH neuron from initiating aversive behavior. Octopamine is an invertebrate biogenic amine that is, structurally, closely related to norepinephrine (Roeder et al. 2003). The group found that octopamine exerts this negative control in the ASH neuron via three alpha-adrenergic-like octopamine receptors: OCTR-1, SER-3, and SER-6 (Mills et al. 2012). Interestingly, this group also identified a peptidergic pathway that exerts a positive effect on ASH activity. Cell-specific RNAi knockdown determined that *C. elegans* aversive response requires a suite of peptides encoded by the neuropeptide, *nlp*-*3. nlp*-*3* signals via the receptor NPR-17 and acts in coordination with a suite of G alpha genes that overlap with octopamine signaling (Harris et al. 2010). To summarize, these studies describe in impressive detail the manner that a sensory circuit is modulated by multiple monoaminergic and peptidergic signaling pathways. Their findings provide many candidate genes and interactions to explore for mammalian avoidance behavior.

## Serotonin signaling in *D. melanogaster* aggression

One of the key elements of fear/anxiety behavior in the vertebrate is the ability to appropriately respond to an external threat. One aspect of a threat response, aggression, particularly in the context of a threat from a con-specific, has been modeled in a simple invertebrate, *Drosophila*. Not surprisingly, serotonin plays an integral role in modulating this behavior.

The fly brain is composed of multiple cell clusters, many of which include varicosities containing serotonin. A complete account of serotonin positive neurons was first mapped in *Drosophila* (Valles and White 1988) using immunocytochemical techniques that were originally used to identify serotonin circuits in non-*Drosophila* insects (e.g., grasshoppers, cockroaches) (Bishop and O’Shea 1983; Nassel and Klemm 1983; Taghert and Goodman 1984). Though the numbers of *Drosophila* serotonin positive neurons identified represent a small fraction of the total neuronal population, their arborization pattern indicates a crucial role for serotonin. The larval serotonin (5-HT) pattern consists of 84 neurons distributed in clusters of one to five neurons labeled SP1, SP2, IP, LP1, LP2, SE1, SE2, SE3, T1, T2, T3, A1-7, and A8 (Valles and White 1988). Further, these clusters are arranged across the two brain hemispheres and the projections fasciculate around the central commissure and innervate the contralateral brain hemisphere (Valles and White 1988; Lundell and Hirsh 1994). In the periphery, 5-HT positive neurons project to the pharynx, proventriculus, the midgut, and the ring gland (larval endocrine organ). In the adult, two new 5-HT clusters (LP2A and LP2B) are added and these are located in the brain near the medulla neuropil. 5-HT is also found in the ellipsoid body of the central complex, all neuropil regions of the optic ganglia, the thoracic ganglia, and the fused abdominal ganglia (Monastirioti 1999). Interestingly, in *Drosophila* and likely other animals, 5-HT is found in a number of neurons that cannot synthesize 5-HT (Valles and White 1988). This result suggests that serotonin re-uptake machinery is expressed in non-serotonin producing cells allowing this key neuromodulator to influence additional circuits (circuits not identified by 5-HT immunoreactivity).

Similar to the vertebrate (Fig. 2b), serotonin is synthesized by two tryptophan hydroxylases and packaged into vesicles by DVMAT (Neckameyer and White 1992; Greer et al. 2005; Neckameyer et al. 2007). Serotonin can activate four receptor subtypes with distinct expression patterns and signaling properties. These receptors are homologous to mammalian receptors in regard to sequence and downstream activity. 5-HT_1A_Dro and 5-HT_1B_Dro are homologues of mammalian 5HT_1_ and are also positively coupled to adenylate cyclase (Witz et al. 1990; Gerhardt et al. 1996). 5-HT_2_Dro is similar to mammalian 5-HT_2_ and activates phospholipase C (Saudou et al. 1992; Colas et al. 1995). 5-HT_7_Dro is functionally similar to vertebrate 5-HT_7_ and upon activation increases cAMP signaling (Saudou et al. 1992). Finally, a serotonin transporter, dSERT, has been identified that has strong sequence and functional homology to the mammalian SERT. This transporter functions to remove serotonin from the synaptic cleft and is highly sensitive to cocaine and anti-depressants (Corey, Quick et al. 1994; Demchyshyn, Pristupa et al. 1994).

This conserved signaling machinery and powerful genetics make *Drosophila* a suitable model to dissect serotonin-mediated behavior. As mentioned, we focus our discussion to one such behavior, a fighting response to an environmental stressor. *Drosophila* exhibits aggressive behavior (Jacobs 1978), which is ethologically (Skrzipek et al. 1979; Lee and Hall 2000) and evolutionarily well established (Boake and Hoikkala 1995; Boake et al. 1998). Male flies under appropriate conditions will occupy a food patch and defend it against other males (Hoffman and Cacoyianni 1989; Hoffman and Cacoyianni 1990). This observation has been transformed into a behavioral assay where pairs of 5- to 7-day-old male flies are placed together in a small chamber (1.6 cm diameter, 1.1 cm height). A careful filming of these encounters found unambiguous offensive fighting elements including wing threat, lunging, holding, tussling, and boxing (Dierick and Greenspan 2007). Interestingly, serotonin pharmacology has been found to play an important role in mediating this aggressive fighting behavior. Flies exposed to 5-HTP, a serotonin precursor, experience a maximum increase in serotonin (5-HT) expression 3 days after treatment, while αMTP, an inhibitor, blocked 5-HT signaling 4 days after exposure. Elevating 5-HT pharmacologically increases aggression in flies, while silencing the serotonergic circuits makes the flies behaviorally unresponsive to the drugs (Dierick and Greenspan 2007). This study was further extended by testing the acute modulation of 5-HT neurotransmission (Alekseyenko et al. 2010). Expression of temperature-sensitive dTrpA1 channels allowed acute activation of serotonergic neurons, which caused flies to escalate fights faster and at higher intensity. In contrast, disruption of 5-HT signaling interferes with the male’s ability to fight and create a dominance relationship (Alekseyenko et al. 2010).

Behavioral traits, such as aggression, are biologically complex, and therefore, establishing a connection between the *Drosophila* aggression phenotype and its underlying genotype often necessitates an understanding of how specific genes orchestrate the development and physiology of the neural circuits that govern the behavioral action (Siwicki and Kravitz 2009). As mentioned above, *Drosophila* aggression is a male-specific phenotype, which suggests aggression is controlled by a sexually dimorphic neural circuit. Ongoing work from the Kravitz laboratory explores how genetic interactions in certain neurons specify the development of these sexually dimorphic neural circuits. *fruitless (fru),* a zinc-finger-like transcription factor, is a part of the sex determination hierarchy of genes in *Drosophila* that control both morphological and behavioral sexual dimorphisms (Siwicki and Kravitz 2009). Transcripts of *fru* undergo complex sex-specific splicing early in *Drosophila* development, ultimately yielding a male-specific *fru* isoform, Fru^M^, and a female-specific *fru* isoform, Fru^F^. The expression of Fru^M^ is necessary and sufficient to specify the neural systems that generate the male patterns of aggressive behavior (Demir and Dickson 2005; Vrontou et al. 2006). Interestingly, a distinctive group of large serotonergic neurons at the posterior tip of the abdominal ganglion (SAbg neurons) express multiple versions of the Fru^M^ isoform (Lee and Hall 2001; Lee et al. 2001). These serotonergic neurons innervate the male reproductive organs. The presence of multiple Fru^M^ isoforms complicates analysis, but the implication is that different combinations of *fru* gene products specify particular components of the neural circuitry concerned with sexually dimorphic behaviors; then, once developed, this circuit can be modulated by serotonergic transmission to effect aggression (Siwicki and Kravitz 2009).

Of course, further cell-specific experiments are needed to define serotonin’s contribution to the neural circuits that drive aggression. We expect the identity of these serotonergic neurons coupled with genetic marking tools (e.g. MARCM) should reveal additional modulators of the aggression circuitry, including but not limited to peptidergic or other monoaminergic pathways. Together, these studies highlight the importance of serotonin signaling and position *D. melanogaster* as a model to study the role of serotonin in cognitive processes relevant to the mammalian brain.

## Conclusions

Human emotional behaviors are generated by a multitude of biological processes. Many of these processes are contingent on the unique neuroarchitecture of complex human cognition and physiology. As stated, one could never accurately model the entire panoply of a human emotional behavior, like anxiety, in an organism that lacks human-like emotional and cerebral capacity. However, we speculate that it is possible to reduce a human emotional behavior down to its component parts and analyze the biological machinery governing individual components using a simpler genetic model. Of course, this analysis is only relevant if the biological machinery in question is conserved between humans and the model organism.

Three interrelated processes help govern human anxiety: the HPA axis, amygdala processing, and serotonin neuromodulation. Neuroanatomical ensembles (HPA axis and amygdala) are vertebrate specific and, therefore, are not candidates for analysis using invertebrate model organisms. However, serotonergic transmission *is* remarkably well conserved between *D. melanogaster*, *C. elegans,* and *H. sapiens* (Fig. 2), despite approximately 550 million years of independent evolutionary trajectories (Valentine et al. 1999). This conservation allows the possibility of utilizing nematodes and flies, with their tractable nervous systems, to explore the specifics of serotonin circuitry as it relates to complex behavioral outputs.

Serotonin signaling certainly modulates affective disorders (i.e., anxiety) in mammals and, as described, also modulates aversive behavior and aggression in invertebrate model organisms. Aversion and aggression do not serve as complete models of anxiety, but rather represent components of this complex human behavior. By observing aversion and aggression in model systems while simultaneously perturbing the genetic/cellular components of the serotonin pathway, one can begin to resolve the complexity of this neurotransmitter’s actions and its intricate interactions with additional signaling pathways. This level of analysis is currently unavailable to mammalian systems, and results from the invertebrate models have the potential to inform the broader neuroscience community.

Treating anxiety disorders with selective serotonin reuptake inhibitors has demonstrated efficacy; However, the adverse side effects associated with SSRIs and their habit of acute anxiety-induction suggest that SSRIs have multiple effects on neurological circuits. A circuit level comprehension of serotonin neuromodulation will greatly enhance our ability to understand and treat anxiety-related disorders. As discussed, serotonin circuits in *C. elegans* are controlled at multiple points by cell-specific receptors and further modulated by octopamine and neuropeptide signaling pathways (Harris et al. 2009, 2010). Perhaps similar circuit level interactions are governing serotonin signaling in the mammalian amygdala, as it pertains to anxiety-related behaviors. The amygdala, specifically the lateral amygdala, has been identified as a key anatomical brain region where circuits involved in the processing of threatening stimulus are housed (LeDoux 2000; Kim and Jung 2006). In all mammalian species studied, the lateral amygdala exhibit exceptionally dense serotonergic innervation, largely originating from the dorsal raphe nucleus (Smith and Porrino 2008; Bonn et al. 2012). It is possible that particular serotonin receptors acting in specific neurons can modulate the manner conditioned and unconditioned stimulus are processed (i.e., coupled) within the serotonergic circuits in the amygdala. Intricate interactions between peptidergic (e.g., neuropeptide Y) and monoaminergic systems (e.g., dopamine, norepinephrine) may couple with serotonin signaling (via distinct serotonin receptors) to provide a more dynamic and sophisticated response to environmental stressors. As circuit level details emerge from invertebrate model systems, candidate interactions are identified, which can then be explored within serotonergic circuits of the lateral amygdala in mammalian systems. We propose that further circuit level analysis of serotonin-mediated behaviors in invertebrate systems will increase our comprehension of serotonin-influenced fearful and anxiety-related behaviors in humans, such as PTSD, phobias, and panic attacks.
